# Fluid Balance, Sodium Losses and Hydration Practices of Elite Squash Players during Training

**DOI:** 10.3390/nu15071749

**Published:** 2023-04-03

**Authors:** Ollie Turner, Nigel Mitchell, Alan Ruddock, Alison Purvis, Mayur K. Ranchordas

**Affiliations:** 1 Academy of Sport & Physical Activity, Sheffield Hallam University, Sheffield S10 2BP, UK; a.ruddock@shu.ac.uk (A.R.); m.ranchordas@shu.ac.uk (M.K.R.); 2 English Institute of Sport, Manchester M11 3BS, UK; nigel.mitchell@eis2win.co.uk

**Keywords:** hydration, fluid, sweat rate, squash, racket sport, sport nutrition, sport physiology, salt, sodium

## Abstract

Elite squash players are reported to train indoors at high volumes and intensities throughout a microcycle. This may increase hydration demands, with hypohydration potentially impairing many key performance indicators which characterise elite squash performance. Consequently, the main aim of this study was to quantify the sweat rates and sweat [Na^+^] of elite squash players throughout a training session, alongside their hydration practices. Fourteen (males = seven; females = seven) elite or world class squash player’s fluid balance, sweat [Na^+^] and hydration practices were calculated throughout a training session in moderate environmental conditions (20 ± 0.4 °C; 40.6 ± 1% RH). Rehydration practices were also quantified post-session until the players’ next training session, with some training the same day and some training the following day. Players had a mean fluid balance of −1.22 ± 1.22% throughout the session. Players had a mean sweat rate of 1.11 ± 0.56 L·h^−1^, with there being a significant difference between male and female players (*p* < 0.05), and a mean sweat (Na+) of 46 ± 12 mmol·L^−1^. Players training the following day were able to replace fluid and sodium losses, whereas players training again on the same day were not. These data suggest the variability in players hydration demands and highlight the need to individualise hydration strategies, as well as training prescription, to ensure players with high hydration demands have ample time to optimally rehydrate.

## 1. Introduction

Squash is a high intensity intermittent sport [1], with elite male squash match play shown to exhibit a mean energy expenditure of 4933 ± 620 kJ·h^−1^, a mean heart rate of 92 ± 3% heart rate maximum, and a respiratory exchange ratio of 0.94 ± 0.06 among players [2]. Elite squash players’ training sessions aim to replicate the high intensity demands of the sport to prepare players appropriately for the rigours of match play [3]. Elite squash players are purported to have high training loads, often engaging in more than one training session per day, spending a total training time greater than 12 h per week, with much training eliciting heart rates greater than 90% heart rate maximum [3,4]. Consequently, these high training loads may increase the hydration demands of players, as there is less time to rehydrate from one session to the next. Moreover, most of these training sessions take place indoors. High exercise intensities, as experienced during elite squash players’ training sessions [3,4], elevate the body’s core temperature [5]. Indeed, 39 min of competitive match play among national standard squash players has been shown to increase core temperature by approximately 1–2 °C (~37 °C to ~39 °C) [6]. As a result, the body has heat loss mechanisms, such as increased skin blood flow and the onset of sweating, to enable evaporative heat loss, maintain heat balance and attenuate further increases in body temperature [7]. 

Hypohydration is defined as a body water deficit greater than an individual’s daily fluctuation [7]. Hypohydration decreases the plasma volume of blood [8], reducing cerebral [9] and muscle blood flow [10], increasing heart rate and cardiovascular strain at any given intensity [11]. Consequently, hypohydration may impair many key performance indicators which characterise elite squash performance [1], such as aerobic capacity [12,13,14,15,16,17,18,19,20,21,22,23,24,25,26,27,28,29,30,31], anaerobic power [32,33,34], muscular endurance [11,32,35,36], lower-body muscular strength [32,37,38,39,40,41,42,43], cognitive function [44,45,46,47,48], and sport specific technical skills [34,49,50,51,52,53].

Accordingly, it is important that squash players have an appropriate hydration strategy to maintain their physical and cognitive performance on court. Developing an optimal hydration strategy is not a one-size-fits-all approach. An athlete’s sweat rate is the main determinant of their hydration strategy [54], with sweat rates being highly individualised due to genetic phenotypes, such as sweat secretion rate per gland [55]. Barnes et al., (2019) [56] found a high variability in athletes’ sweat rates across a variety of sports, such as running, cycling, American football, tennis, and soccer, reporting absolute whole body sweat rates ranging from 0.16 to 5.73 L·h^−1^. As well as fluids, Na^+^ is also an important factor in a player’s hydration strategy. Na^+^ is an electrolyte lost in sweat, which has been shown to maintain plasma levels of vasopressin and aldosterone, promoting whole-body and extracellular fluid retention [57]. Na^+^ containing fluids have been shown to optimise rehydration post-exercise [58]. Like sweat rates, sweat [Na^+^] is also highly individualised, with Ranchordas et al., (2017) [59] reporting sweat [Na^+^] ranging from 11.2 mmol·L^−1^ to 86.5 mmol·L^−1^ among professional male team sport athletes (soccer, rugby, American football, baseball and basketball). Consequently, an individual’s hydration strategy is personalised, depending on the individual, their sweat rate, and their sweat [Na^+^].

Previous research has quantified the fluid balance, sweat [Na^+^] losses and hydration practices of athletes in a variety of racket sports, such as tennis [60,61,62,63] and badminton [64], as well as high intensity intermittent sports, such as soccer [65,66,67,68,69,70,71,72,73,74,75,76,77,78,79], American football [80,81,82,83,84,85,86,87,88], basketball [89,90,91,92], field hockey [93]; ice hockey [94,95,96,97], Gaelic football [98], rugby league [99,100], and rugby union [101,102]. Despite this, no study has quantified the fluid balance, sweat [Na^+^] or hydration practices of elite squash players during a training session. Calculating the fluid balance, sweat [Na^+^] and hydration practices of elite squash players during a training session would provide players, coaches, and practitioners with information to optimise players’ hydration practices throughout training.

Therefore, the primary aim of this study was to quantify the sweat rates and sweat [Na^+^] of elite squash players throughout a training session. A secondary aim was to investigate players’ hydration practices during the training session (i.e., hydration status pre-session, fluid and Na^+^ intake during and post-session) to determine whether these were optimal in relation to a players individualised sweat rate and sweat Na^+^ losses. A final aim of the study was to determine players’ pre-conceived sweat rate, sweat [Na^+^], and cramping frequency, and to quantify the relationship between these, players’ sweat rates, and players’ sweat [Na^+^].

## 2. Materials and Methods

### 2.1. Experimental Design

Data was collected during a training session with an England Squash senior squad in March 2021. This training session was the first of a training microcycle. The training session was consistent with training that would normally be undertaken at an England Squash senior squad and consisted of players’ habitual warm up, squash-specific routines and conditioned games. The training session lasted 90 min (from 10:00 to 11:30). Player’s pre-exercise hydration status was assessed before the session through urine osmolarity; players’ sweat rate, fluid and food intake measured during the session; and players’ sweat [Na^+^] quantified post-session. Players completed a food and drink diary immediately after the session until the time of their next training session, in order to quantify their rehydration practices. 

### 2.2. Participants

Fourteen (males = 7, females = 7) elite (tier 4) and world class (tier 5) [103] squash players (male (age = 25 ± 5 years; stature = 184 ± 2 cm; body mass = 78.9 ± 7.3 kg); female (age = 25 ± 4 years; stature = 169 ± 7 cm; body mass = 63.7 ± 8.6 kg) volunteered to take part in the study. All experimental procedures and risks were explained to the players, with written informed consent being collected before data collection. The research was approved by an institutional ethics committee (ER28778154) and conducted in accordance with the principles of the 7th revision of the Declaration of Helsinki [104].

### 2.3. Fluid Balance

Upon arrival, a pre-exercise urine sample was assessed for urine osmolarity (Vitech Scientific LTD, UK) to quantify the pre-exercise hydration status of players. Values of <700 mOsmol·kg^−1^ were used as a guide for euhydration [54]. Urine osmolarity has been reported to be a valid and reliable instrument to assess athlete’s hydration status [105]. Players’ stature was measured prior to the training session using a stadiometer (SECA, Alpha 213, Hamburg, Germany). Pre- and post-exercise nude body mass was assessed in conjunction with food and fluid balance to quantify whole body sweat loss through the following equation:

$$\begin{array}{l} Total\;sweat\;mass\;loss\\ =Post\;exercise\;body\;mass\;(Nude\;body\;mass\;post\;exercise\\ -Food\;and\;fluid\;intake\;during\;exercise\\ +urine\;and\;stool\;output\;during\;exercise)\\ -Nude\;body\;mass\;pre\;exercise\; \end{array}$$

Players were instructed to collect all urine and stools passed during the training session in pre-weighted containers. There were frequent breaks in the training session whereby players had the opportunity to consume fluid and/or food, as is common practice in squash training sessions. Players were instructed not to squirt or spit fluid from their drink bottles. A Wahoo TickrX (Atlanta, GA, USA) was used alongside the Wahoo mobile application to assess heart rate during the training session. Post-session, players rating of perceived exertion (RPE) was recorded using the CR10 Scale [106] and multiplied by the duration of the session (min) to calculate sRPE [107].

### 2.4. Sweat Sodium Collection and Analysis

Sweat samples were collected according to the methods of Ranchordas et al., (2017) [59]. Players were rested for 5 min prior to the sweat sample being collected. Post the 5-min rest period, the left mid-forearm was cleaned with an alcohol wipe as well as purified water to remove any dirt and sweat from the skin. It was also checked for breaks, fissures, or any inflammation [108]. Two stainless-steel electrodes and iontophoretic discs were then applied to the cleaned area. The iontophoretic discs consisted of a solid agar gel which consisted of 96% water, 0.5% pilocarpine nitrate and trace antifungal compounds. Consequently, sweat was induced to the left mid-forearm where the electrodes and iontophoretic discs were placed, through a 1.5-mA iontophoretic current, lasting for 5 min (Webster Sweat Inducer, Wescor Inc., Logan, UT, USA). The iontophoretic discs were subsequently removed, and the stimulated area cleaned with purified water and blot dried. A tailored plastic disk was applied to the induced area (Macroduct Sweat Collector, Wescor Inc.). An 85 μL sweat sample was then collected via hydraulic pressure. This typically took 10 to 30 min, with samples less than 85 μL discarded. A blunt-fill needle and syringe was used to eject the sweat sample from the collection disk. This passed through a conductivity cell (Sweat Check™, Wescor Inc.), which quantified NaCl molarity from the sweat sample conductivity. The conductivity cell was cleaned and calibrated before each test using deionised water and a dummy sample of a known sodium concentration, respectively.

Pilocarpine iontophoresis has been validated against the Gibson and Cooke Gauze technique [109], as well as displaying strong correlations with flame photometry [110] and qualitative pilocarpine iontophoresis [111]. Pilocarpine iontophoresis has conveyed excellent absolute (CV < 2.6%) and relative reliability (ICC > 0.99) [112].

### 2.5. Post Session Fluid and Sodium Consumption

Players’ post-session fluid (L) and Na^+^ (mg) consumption were calculated through a self-reported food and drink diary. Food and drink diaries were completed via the mobile application WhatsApp (Facebook, Menlo Park, CA, USA), with players sending across details of any food or drink they consumed (i.e., name, mass and cooking methods), as well as an accompanying photo, which was timestamped. The calculation of fluid intake also included any fluids consumed through food consumed. Players were instructed to complete the food and drink diary immediately after training until the time of their next training session. For seven players, this was 14:00 the same day (amount of time in between sessions = 2 h 30 min between session) (same day training session group), whereas, for the other seven players, this was 09:00 the next day (amount of time in between sessions = 21 h and 30 min) (following day training session group). Food and drink diaries were analysed by the principal researcher, who is a Sport and Exercise Nutrition Register (SENr) accredited nutritionist, using the nutrition analysis software Nutritics (Nutritics Ltd, Swords, Ireland).

### 2.6. Perceived Sweat Rate and Sweat Sodium Concentration Measures

During the sweat [Na^+^] collection, participants were asked three questions regarding their perceived sweat rate, frequency of cramps and perceived sweat [Na^+^]. The questions (and answers) were as follows: (1) “How would you classify your sweat rate?” (low, moderate, high, very-high); (2) “How often do you suffer from muscle cramps?” (never, rarely, sometimes, often); (3) “Do you think you lose a lot of Na^+^ in your sweat?” (no, yes). Before answering question three, participants were asked to consider whether they felt sweat irritating their eyes. These questions were asked to quantify whether players’ preconceptions regarding their sweat rate and sweat Na^+^ losses were correct, and whether players altered their hydration strategy accordingly (e.g., if a player preconceives a high sweat rate, are they more likely to consume more fluid during the session). These questions have been shown to provide sufficient reproducibility in identifying relationships between participants sweat rate (ICC = 1.0), frequency of cramps (ICC = 0.9), sweat [Na^+^] perceptions (ICC = 0.7), and quantifiable variables (Ranchordas et al., 2017) [59].

### 2.7. Statistical Analysis

SPSS V 24.0 software (SPSS Inc., Chicago, IL, USA) was used to perform the data analysis. All data was displayed as mean ± standard deviation for all participants, with *p *< 0.05 being the criterion for significance among all statistical tests. The Shapiro-Wilk test was used to assess normal distribution. Levene’s Test for Equality of Variances was used to assess homogeneity of variance. Independent Samples T-Test or Mann-Whitney *U* Test (for non-parametric analysis) was used to analyse the differences in (1) sweat rates between males and females, (2) the differences in sweat Na^+^ losses between males and females, and (3) post-session hydration strategies between the two subsequent training groups (same day training session group vs. following day training session group). Comparison between the same day training session group and the following day training session group were analysed through comparing (1) post-session fluid consumption, and (2) post-session Na^+^ consumption between the two groups. Effect sizes were interpreted according to accepted thresholds (small: *d* = 0.2; moderate: *d* = 0.5; large = 0.8) [113]. Pearson’s Correlation Coefficient or Spearman’s Rank-Order Correlation (for non-parametric analysis) was used to quantify the relationship between (1) players perceived sweat rate and their sweat rate, (2) players’ sweat [Na^+^] and their self-reported cramping frequency, (3) players’ sweat [Na^+^] and their self-reported sweat [Na^+^]. Pearson’s Correlation Coefficient and Spearman’s Rank-Order Correlation were interpreted according to accepted thresholds (very weak: *r* = 0–0.19; weak: *r* = 0.2–0.39; moderate: *r* = 0.4–0.59; strong: *r* = 0.6–0.79; very strong: *r* = 0.8–1) [114].

## 3. Results

### 3.1. Environmental Conditions

Mean temperature throughout the training session was 20 ± 0.4 °C, with mean humidity 40.6 ± 1%.

### 3.2. Pre-Session Urine Osmolarity

**Figure 1:**
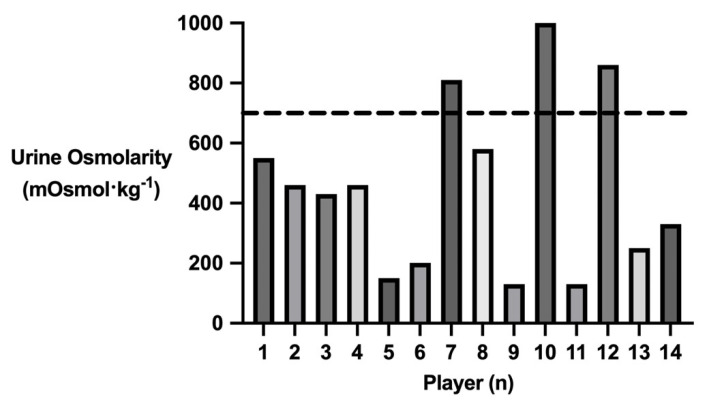
Player’s pre-session urine osmolarity. Dotted line denotes 700 mOsmol·kg^−1^.

Figure 1 conveys players’ pre-session urine osmolarity. Mean pre-session urine osmolarity among players was 453 ± 283 mOsmol·kg^−1^, with individual values ranging from 130 m0smol·kg^−1^ to 1000 mOsmol·kg^−1^. Eleven players (79%) reported a value <700 mOsmol·kg^−1^, indicating euhydration [54], with the remaining three players reporting values >700 mOsmol·kg^−1^, denoting hypohydration [54].

### 3.3. Training Load

Table 1 conveys the training load of each player throughout the training session.

### 3.4. Fluid Balance, Sweat Rate and Sweat Sodium Composition

**Figure 2:**
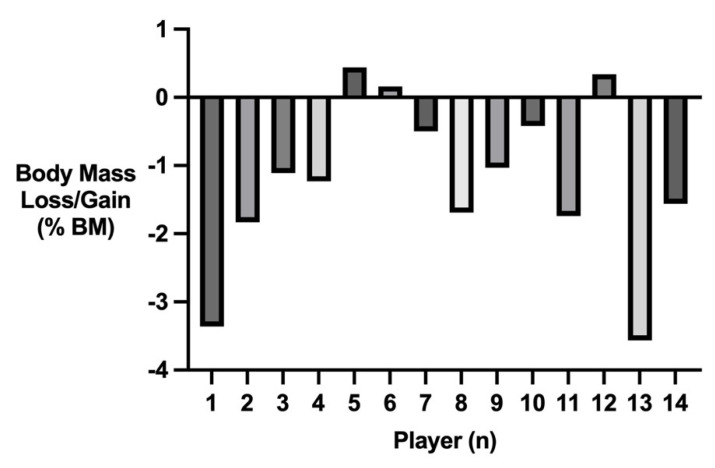
Player’s body mass loss/gain during the training session (% body mass).

Figure 2 conveys the body mass loss/gain of players throughout the training session. Eleven players lost body mass throughout the session, while three players gained mass. The mean body mass loss/gain was −0.91 ± 0.95 kg. This equated to a mean body mass loss/gain percentage of −1.22 ± 1.22%. 

**Figure 3:**
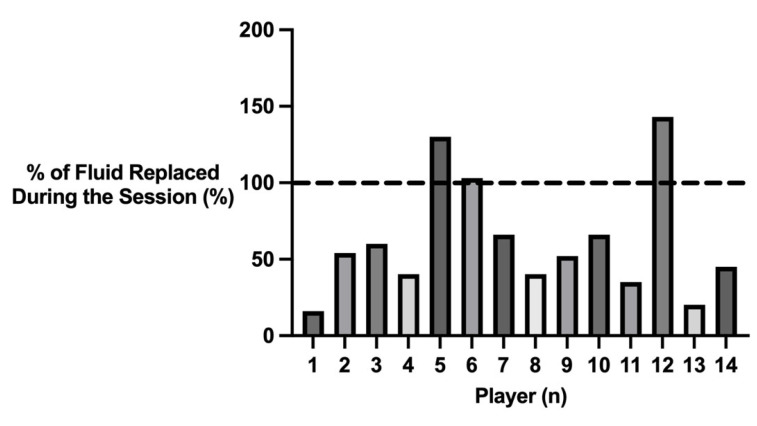
% of fluid replaced by each player during the training session. Dotted line denotes 100% of fluid replaced during the session.

Players had a mean sweat mass loss of 1.66 ± 0.92 kg and mean fluid intake of 0.79 ± 0.31 L throughout the training session. This equated to a mean sweat rate and fluid intake of 1.11 ± 0.56 L·h^−1^ and 0.53 ± 0.21 L·h^−1^, respectively. Figure 3 displays the % of fluid replaced during the training session. Players replaced a mean amount of 62 ± 38% of fluids which were lost during the session. Three players replaced more than 100% of their sweat losses during the session.

**Figure 4:**
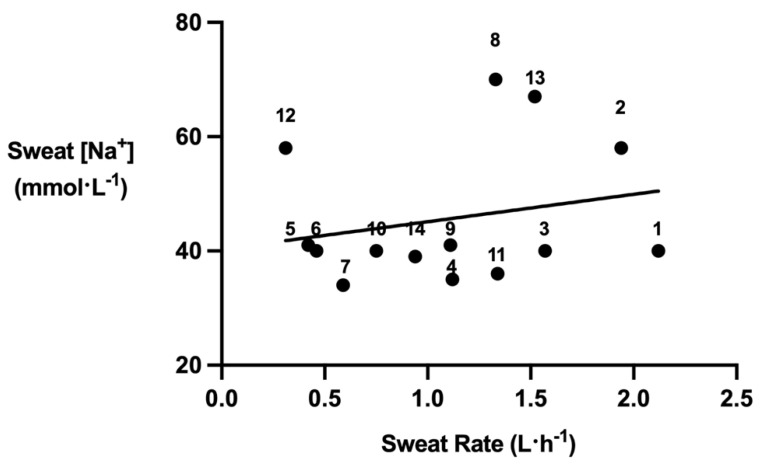
Players’ sweat [Na^+^] losses. Number (n) above the symbol denotes player number.

Figure 4 conveys players’ sweat [Na^+^]. Players mean sweat [Na^+^] was 46 ± 12 mmol·L^−1^. Players had a mean sweat Na^+^ rate of 934 ± 248 mg·L·h^−1^, equating to a mean total Na^+^ loss of 1598 ± 1023 mg throughout the training session.

### 3.5. Differences in Sweat Rates and Sweat (Na+) between Males and Females

Male players had a significantly greater sweat rate in comparison to female players (males = 1.47 ± 0.45 L·h^−1^; females = 0.78 ± 0.45 L·h^−1^; *p* = 0.01; *d* = 1.53; CI = 0.16–1.22). There were no significant differences in sweat [Na^+^] (males = 46 ± 12 mmol·L^−1^; females = 44 ± 12 mmol·L^−1^; *p* = 0.53; *d* = 0.16; CI = −13.09–16.23).

### 3.6. Post Session Fluid and Na^+^ Intake

**Figure 5:**
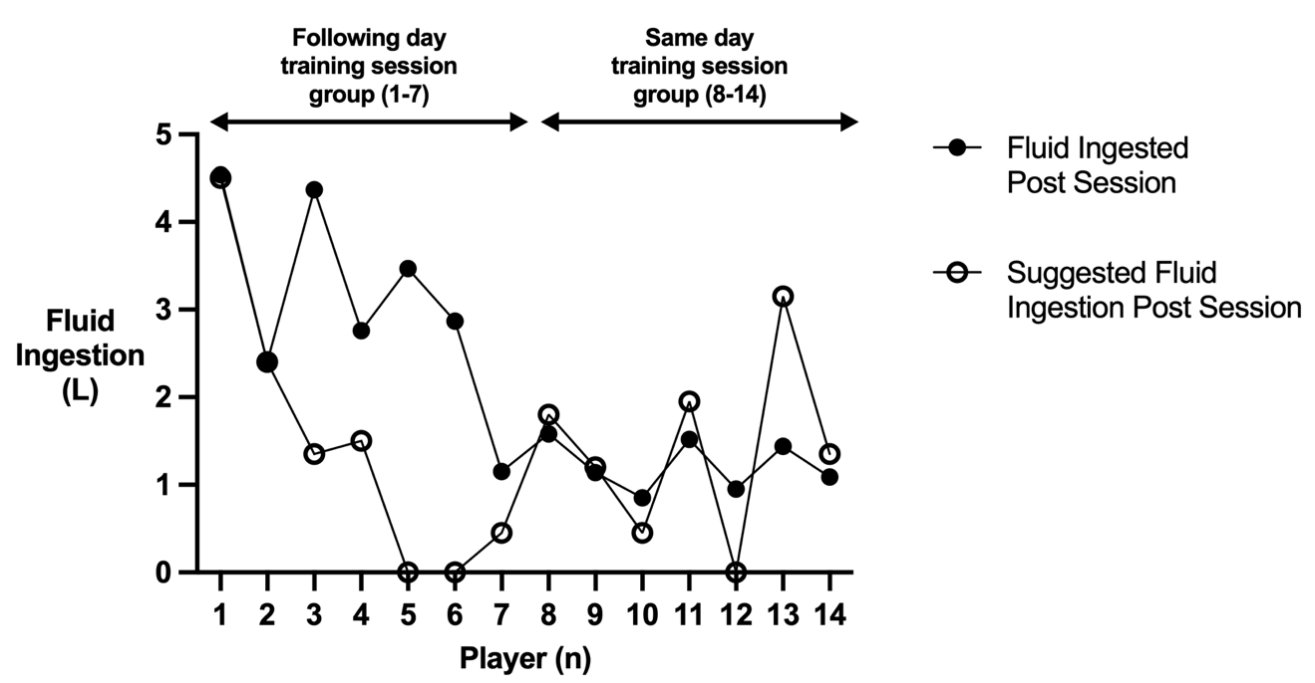
Players post session fluid ingestion in relation to their suggested post session fluid ingestion.

**Figure 6:**
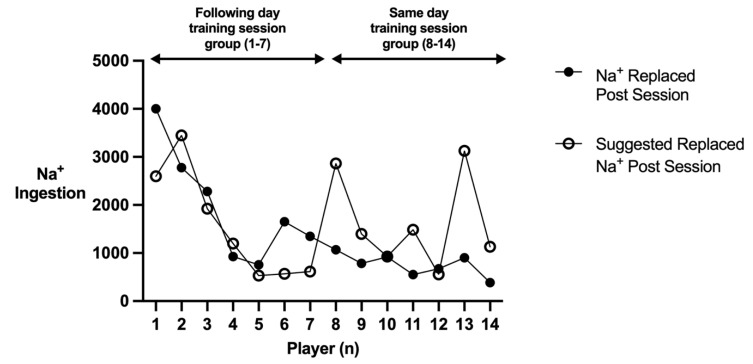
Players post session Na^+^ ingestion in relation to their suggested post session Na^+^ ingestion.

Figure 5 conveys the post-session fluid intake in relation to suggested post-session fluid intake [54], while Figure 6 displays the post-session Na^+^ intake in relation to suggested post-session Na^+^ intake [54]. There was a significant difference between post-session fluid (*p* = 0.002; *d* = 2.2; CI = 0.859–2.848) and post-session Na^+^ intake (*p* = 0.011; *d* = 1.46; CI = 242.6–2174.8) in the following day training session group and the same day training session group. All seven players in the following day training session group replaced 1.5 L of fluid for every kilogram of body mass lost throughout training, with five replacing the amount of Na^+^ lost during the session. Only two out of the seven players in the same day training session group managed to replace 1.5 L of fluid for every kilogram of body mass lost throughout the training session, with only one player managing to replace the amount of Na^+^ lost.

### 3.7. Relationship between Players Perceived Sweat Rate and Sweat (Na+)

There was a moderate positive association between players perceived sweat rate (Q1) and sweat rate (*r* = 0.51; *p* = 0.62), a very weak positive association between perceived incidence of cramps (Q2) and sweat [Na^+^] (*r* = 0.01; *p* = 0.97), and a very weak negative association between perceived sweat [Na^+^] (Q3) and sweat [Na^+^] (*r* = −0.02; *p* = 0.95).

## 4. Discussion

The primary aim of this study was to quantify the fluid balance, sweat rates and sweat [Na^+^] of elite squash players during a training session. The secondary aims were to (1) quantify the differences in sweat rates and sweat [Na^+^] between male and female elite squash players; (2) calculate players hydration practices during the training session to determine whether these were optimal in relation to players’ sweat rate and sweat Na^+^ losses; (3) to establish whether players who have two training sessions on the same day, have sufficient time to appropriately rehydrate in comparison to players who are next training the following day; (4) to determine players perceived sweat rate, sweat [Na^+^] and cramping frequency and to quantify the relationship between these and players’ sweat rates and sweat [Na^+^].

The main findings were (1) elite squash players had a mean fluid balance of −1.22 ± 1.22% throughout the session; (2) elite squash players had a mean sweat rate of 1.11 ± 0.56 L·h^−1^; (3) elite squash players had a mean sweat [Na^+^] of 46 ± 12 mmol·L^−1^; (4) males had a significantly greater sweat rate than females; (5) players in the same day training session group were not able to replace fluid and sweat Na^+^ losses, whereas players in the following day training session group were able to do so; (6) there was a moderate positive, but non-significant association between players perceived sweat rate and their sweat rate.

### 4.1. Pre-Session Hydration Status

Pre-session urine osmolarity conveyed that 11/14 players (79%) commenced the training session euhydrated (<700 mOsmol·kg^−1^) [54], with a mean pre-session urine osmolarity of 453 ± 283 mOsmol·kg^−1^. Previous research investigating the pre-exercise hydration status of athletes in a variety of sports is equivocal, with some athletes displaying euhydration pre-exercise [65,68,70,71,73,75,77,79,80,90,93,96,97,98,100,102,115] and others displaying hypohydration pre-exercise [62,63,66,72,74,75,76,78,79,80,82,84,88,89,91,92,94,95,101,116]. Athletes’ hydration status appears to be individualised, as exhibited by the range in values recorded in the present study (150 mOsmol·kg^−1^ to 1000 mOsmol·kg^−1^). Indeed, previous research displays high variability among cohorts of athletes [62,63,65,66,68,70,71,72,73,74,75,76,77,78,79,80,82,84,88,89,90,91,92,93,94,95,96,97,98,100,101,115,116].

The present data was collected at the start of a training micro-cycle, whereby players may have had ample opportunity to ensure euhydration prior to the session. Godek et al., (2005) [80] reported American Collegiate Football players to be in a euhydrated state at the start of a training week (urine specific gravity = 1.017 ± 0.06), but experienced hypohydration from training days two to eight (urine specific gravity => 1.020). Future research should aim to quantify the hydration status of elite squash players throughout a micro-cycle, and whether there is a compounding effect of a player’s training demands on hydration status.

### 4.2. Fluid Balance

Elite squash players had a mean sweat rate of 1.11 ± 0.56 L·h^−1^ during the training session. To our knowledge, no other racket sport has quantified the sweat rates of players throughout a training session, making it difficult to compare. Abian et al., (2012) [64] and Lott and Galloway [63] quantified the sweat rates of elite badminton players and university tennis players through match play, when performed indoors at moderate environmental conditions (temperature = 17–24 °C; humidity 40–60%), respectively. They reported similar sweat rates to the present data, with elite badminton players shown to have a sweat rate of 1.08 ± 0.53 L·h^−1^ and tennis players a sweat rate of 1.10 ± 0.4 L·h^−1^. Environmental conditions of sports performed indoors can be regulated, and therefore players intraindividual sweat rates are primarily influenced by the intensity of effort, the clothing they wear, and their hydration status [117]. Research has shown that elite squash match play may be performed at a higher intensity in comparison to training sessions [2,3,4]. Consequently, future research should aim to quantify the sweat rates of elite squash players during match play to quantify differences, in comparison to training. This would also allow for greater comparison against other racket sports as this data has previously only been collected during match play [63,64].

It is difficult to compare elite squash players’ sweat rates to high intensity intermittent sports performed outdoors, due to differences in environmental conditions. Maughan et al., (2004) [65] reported elite soccer players’ sweat rates to be 1.4 ± 0.3 L·h^−1^ during a training session, although this was performed at a wet globe temperature of 26.6 °C and relative humidity of 54.8%. Environmental conditions have been shown to influence sweat rates [117]. Rollo et al., (2021) [78] reported that elite male soccer players had a higher sweat rate when performing high intensity training in hot environmental conditions (WGBT = 29 ± 1 °C; RH = 52 ± 7%; sweat rate = 1.43 ± 0.23) in comparison to cool environmental conditions (WGBT = 15 ± 7 °C; RH = 66.6%; sweat rate = 0.98 ± 0.21), due to an increase in the contribution of evaporative requirement for heat balance [117,118]. Elite squash tournaments are played all around the world in a variety of environmental conditions [119]. This includes hot conditions, such as when the 2022 Professional Squash Association World Championships were staged outdoors in Cairo, Egypt [120]. Consequently, future research should quantify the sweat rates of elite squash players during hot conditions to compare differences to moderate environmental conditions (temperature = 17–24 °C; humidity 40–60%). This would help players ascertain whether different hydration strategies are required when competing in hot and humid environmental conditions.

The present study highlights the interindividual variation in sweat rates among players. Sweat rates ranged from 0.31 to 2.12 L·h^−1^, conveying that a player’s hydration strategy is not a one size fits all approach. This is consistent with data collected by Barnes et al., (2019) [56], who, despite reporting a mean whole body sweat rate of 1.13 ± 0.58 L·h^−1^ among a variety of different team & skill sport athletes, conveyed a sweat rate range of 0.16 to 5.73 L·h^−1^. Interindividual variations in sweat rates may be due to genetic phenotypes of an individual’s sweat glands, such as the size of the gland and its methacholine sensitivity [121,122].

Players consumed a mean fluid intake of 0.53 ± 0.21 L·h^−1^ during the training session. This is reported to be less than elite soccer players through training (0.97 ± 0.3 L·h^−1^) [65], elite badminton players during match play (1.08 ± 0.5 L·h^−1^) [64], and university tennis players throughout match play (1.09 ± 0.63 L·h^−1^) [63]. The lower fluid intakes of elite squash players could be due to individuals not wanting to consume high volumes of fluid while performing squash specific movements such as lunges and changes of direction [123], or alternatively, there may not be the opportunity to consume fluids as in other high intensity intermittent sports. The opportunities for fluid consumption of elite squash players during training are normally unscheduled and intermittent breaks in play at the coaches’ discretion. This contrasts with competitive match play, whereby players are allowed 120 s at each game interval before the next game starts. Thus, future research should also aim to quantify whether this alters the fluid consumption dynamics in comparison to training. 

Players replaced 62 ± 38% of fluid lost during the training session, with three players consuming more than 100% of their sweat losses during the session. Consequently, players had a mean body mass loss/gain of −0.91 ± 0.95 kg, equating to a mean body mass loss/gain percentage of −1.22 ± 1.22%. This is a greater body mass loss in comparison to elite badminton players (−0.35 ± 0.67%) [64] and university tennis players (−0.15 ± 0.79%) [64]. This appears to be due to the lower volumes of fluid consumed during squash. The present study conveys the variability in fluid balance among elite squash players, highlighting the need for an individualised approach to hydration. Three players increased body mass during the session, while two players reported a body mass loss greater than 2%, the associated threshold for a decline in physical and cognitive performance [54]. Previous research has quantified the effects of hypohydration on sport-specific performance in basketball [44,51,52], cricket [50,53], field hockey [46], golf [47], horse racing [38], and soccer [49]. Future research should aim to quantify if, and at what level, hypohydration decreases squash-specific performance [1].

### 4.3. Sweat (Na+) Losses

Elite squash players had a mean sweat [Na^+^] of 46 ± 12 mmol·L^−1^. This is comparable to data collected by Ranchordas et al., (2017) [59], who conveyed a mean sweat [Na^+^] of 44 ± 13 mmol·L^−1^ across a variety of different sports (soccer, rugby, American football, baseball and basketball), utilising the same technique (pilocarpine iontophoresis). The present data equated to a mean sweat Na^+^ rate of 934 ± 248 mg·L·h^−1^, and a mean total Na^+^ loss of 1598 ± 1023 mg throughout the training session. Na^+^ can be easily consumed in the diet through adding NaCl (i.e., table salt) to meals or sport hydration/electrolyte tablets to water (most commercially available ones contain ~400–1000 mg Na^+^). Consequently, players should aim to consume approximately 1000 mg of Na^+^ per hour of squash training to replace Na^+^ losses. 

The present study exhibited high variability in sweat [Na^+^], ranging from 34 mmol·L^−1^ to 70 mmol·L^−1^. Therefore, while this recommendation of 1000 mg of Na^+^ per hour of squash training may be advisable for players with moderate sweat [Na^+^], players with a high sweat [Na^+^] may require a more aggressive strategy to replace sweat Na^+^ losses. Variability in sweat [Na^+^] may be due to heat acclimation, through an increased Na^+^ ion reabsorption capacity of the eccrine sweat gland [124,125], as well as the sweat glands increased sensitivity to aldosterone [126] or differences in dietary Na^+^ intake [127,128].

### 4.4. Differences between Males and Females

Male players had a significantly greater sweat rate in comparison to female players (*p* = 0.015). This is consistent with findings from Barnes et al., (2019) [56], who found that males had a significantly greater sweat rate than females during a variety of different sports and intensities. Differences in sweat rate between males and females could be due to fluctuations in the female sex hormones, estrogen and progesterone [129,130]. Transcapillary fluid dynamics have been reported to be altered as a result of differing levels of estrogen and progesterone, influencing intracellular and extracellular fluid balance [131]. Female sex hormones may also influence thresholds for synthesis and release of arginine vasopressin, a volume regulatory hormone [132], as well as influencing fluid regulatory dynamics by increasing interstitial fluid during the luteal phase [131]. These physiological changes at various time points throughout a female player’s menstrual cycle may influence sweat rate, increasing water retention [133].

There were no significant differences between males and females in sweat [Na^+^] (*p* = 0.53). This is consistent with Barnes et al., (2019) [56], who found no significant differences between male and female athletes across a variety of different sports. Female sex hormones influence on net Na^+^ balance is equivocal [130], and variations in sweat [Na^+^] are likely to be due to interindividual and intraindividual differences [117], as previously discussed (see Section 4.3).

### 4.5. Post-Session Fluid and Na^+^ Consumption

Current ACSM guidelines recommend consumption of 1.5 L of fluid for every kilogram of body mass lost during exercise as well as replacing any Na^+^ lost [54]. All seven players in the following day training session group replaced 1.5 L of fluid for every kilogram of body mass lost throughout the training, with five replacing the amount of Na^+^ lost during the session. Only two of the seven players in the same day training group managed to replace 1.5 L of fluid for every kilogram of body mass lost throughout the training session, with only one player managing to replace the amount of Na^+^ lost. Players in the following day training session group had 21 h and 30 min to rehydrate, whereas players in the same day training session group had 2 h and 30 min to complete the same rehydration. Consequently, differences in fluid and Na^+^ consumption post-session could be due to a lack of time in-between training sessions, poor time management, and/or logistical issues (e.g., fluids are not available for players to consume). As a result, players should work with sports science practitioners and coaches to devise their training schedule and training load with consideration to their hydration demands. Players should also ensure that they are adequately prepared according to their hydration demands (e.g., have enough fluids and appropriate Na^+^ containing foods) to ensure they optimally rehydrate in between sessions. Squash players often engage in more than one training session per day [3,4]. Future research should aim to track the hydration status alongside body mass and hydration practices throughout squash players’ microcycle, in order to determine whether there is a compounding effect of training on players’ hydration status.

### 4.6. Players’ Perceived Sweat Rate, Sweat (Na+) and Perceived Incidence of Cramps

There was a moderate positive association between perceived sweat rate and sweat rate. This is consistent with findings in male and female division II basketball players during practice, who were able to estimate sweat losses in relation to actual sweat losses during sport-specific practice (*r* = 0.87; *p* < 0.001) [91]. Sweat rates may be easy for squash players to determine due to visual cues, such as the amount of sweat on apparel or on the court while playing.

Players were shown to perceive a very weak negative association between their perceived sweat [Na^+^] and their sweat [Na^+^]. This suggests that players could not predict how much Na^+^ they lost in their sweat, and contrasts with findings from Ranchordas et al., (2017) [59] of a strong positive correlation between athletes’ sweat [Na^+^] and self-reported [Na^+^] (*r* = 0.82; *p* < 0.001). [Na^+^] in sweat may be more difficult to predict, as there is no visual stimulus, unlike sweat rates, aside from Na^+^ residue on apparel, which is only visible in darker coloured clothing. 

Players also conveyed a very weak positive association between perceived incidence of cramps and sweat [Na^+^]. This is consistent with findings from Ranchordas et al., (2017) [59] who found the same weak positive correlation (*r* = 0.18; *p* = 0.015). The causes of cramp are ambiguous and therefore a causative relationship between cramps and electrolyte depletion is equivocal [134].

### 4.7. Limitations

There are a few limitations to the present study. Firstly, players may have been aware that their hydration status was being assessed prior to the training session. Players were partially blinded, as they were asked to provide a urine sample prior to the training session without clarification as to why. However, some players may have guessed that this was to quantify hydration status and altered hydration practices pre-session accordingly (i.e., consumed more fluids to ensure they were euhydrated). Urine osmolarity is also reported to lack clinical sensitivity in comparison to blood tonicity when measuring hydration status [135]. Despite this, urine osmolarity has been reported to be a reliable and practical instrument in assessing athlete’s hydration status in the field [105].

Another potential limitation is the technique used to measure sweat [Na^+^]. Pilocarpine iontophoresis is a regional sweat collection technique, with the present study utilising a player’s forearm to quantify sweat [Na^+^]. Taylor et al., (2013) [136] reported variations in regional sweat [Na^+^], with regional sweat [Na^+^] techniques also shown to overvalue sweat [Na^+^] in comparison to whole body sweat [Na^+^] [137] (whole body Na^+^ loss = 51.6 ± 18.3 mM; regional Na^+^ loss = 75.3 ± 21.9 mM). However, due to the nature of the squash, the whole body sweat [Na^+^] technique proposed by Shirreffs & Maughan (1997) [137] is not plausible. Other research [138,139] has collected samples from various sites of the body (e.g., forearm, quadricep etc.), and ran either a regression equation or combined measures into an arithmetic mean to develop a measure of whole body sweat [Na^+^]. However, these regression equations are derived from sweat-patch tests, which may compromise the validity of their results [59]. The local sweat response to pilocarpine iontophoresis is proposed to differ in comparison to exercise induced sweating, due to differences in the sudomotor response [117,140]. Exercise-induced sweating is modulated through a variety of local and central mediators, such as skin blood flow and exercise pressor reflex [141,142]. Despite this, differences in sweat [Na^+^] composition between pharmacologically induced sweat (i.e., pilocarpine iontophoresis) and thermally induced sweat (i.e., during exercise) are equivocal, with some studies reporting pharmacologically induced to have greater sweat [Na^+^] and some reporting similar sweat [Na^+^] [143,144]. Consequently, as the techniques employed in the present study are valid and reliable (see Section 2.4), we opted for pilocarpine iontophoresis.

Finally, another limitation of this study is that we did not quantify female players’ hormonal status to better understand how this may influence their sweat rate. Female sex hormones may influence female players’ sweat rates (see Section 4.4).

### 4.8. Practical Applications

This study highlights that it is possible to collect hydration-related data in elite squash players and conveys a framework to determine them. It also displays the variability in hydration demands across players and emphasises the importance of individualised hydration strategies, rather than a one-size-fits-all approach. For example, player 1 had a sweat rate of 2.12 L·h^−1^ and sweat [Na^+^] of 40 mmol·L^−1^, losing 3.17 L of fluid and 2589 mg of Na^+^ throughout the session. This contrasts with player 5, who had a sweat rate of 0.42 L·h^−1^ and sweat [Na^+^] of 41 mmol·L^−1^, losing a total fluid and Na^+^ loss of 0.63 L and 530 mg respectively. Consequently, the hydration strategies employed with each player may be different, even though the training loads may be the same. Player 5 had a low hydration demand, and fluid and Na^+^ losses can be accounted for throughout or immediately post-session. This is unlike player 1, who had a high hydration demand, with it being unlikely to replace sweat and Na^+^ losses during the session, requiring an aggressive rehydration strategy post-session, as well as consideration of the proximity of the next training session undertaken.

## Figures and Tables

**Table 1:** Player’s training load throughout the training session.

Participant (n)	Sex (M/F)	Duration (min)	Mean Heart Rate (Beats.min^−1^)	Mean % of Age Predicted Heart Rate Maximum (%)	Maximum Heart Rate (Beats.min^−1^)	Maximum % of Age Predicted Heart Rate Maximum (%)	RPE (*n*/10)	sRPE (AU)
1	M	90	153	78	190	97	7	630
2	M	90	151	77	185	94	7	630
3	M	90	149	75	189	95	6	540
4	F	90	144	76	188	99	5	450
5	F	90	132	69	173	91	5	450
6	F	90	162	81	189	95	7	630
7	F	90	131	68	175	91	6	540
8	M	90	157	82	191	100	6	540
9	M	90	145	78	178	96	4	360
10	M	90	124	63	161	81	7	630
11	M	90	157	79	184	92	6	540
12	F	90	158	79	185	93	7	630
13	F	90	121	61	161	82	3	270
14	F	90	162	82	191	96	4	360
Mean ± SD	-	90	146 ± 14	75 ± 7	181 ± 10	93 ± 6	6 ± 1	514 ± 119

## Data Availability

Most of the data generated or analysed during this study are included in this published article such as individual sweat rates, sweat (Na+) etc. Full individual data cannot be request due to identification of players (such as identification through age and body mass etc.).
